# Identification of Novel and Recurrent Variants in *BTD*, *GBE1*, *AGL* and *ASL* Genes in Families with Metabolic Disorders in Saudi Arabia

**DOI:** 10.3390/jcm13051193

**Published:** 2024-02-20

**Authors:** Muhammad Latif, Jamil Amjad Hashmi, Abdulfatah M. Alayoubi, Arusha Ayub, Sulman Basit

**Affiliations:** 1 Department of Basic Medical Sciences, College of Medicine, Taibah University, Madinah 42353, Saudi Arabia; jamjadali@taibahu.edu.sa (J.A.H.); aayoubi@taibahu.edu.sa (A.M.A.); 2 Center for Genetics and Inherited Diseases, Taibah University, Madinah 42353, Saudi Arabia; 3 Department of Medicine, School of Health Sciences, University of Georgia, Tbilisi, P. O. Box-0171, Georgia; arushaayub.std@ug.edu.ge

**Keywords:** metabolic disorder, *BTD* mutation, *ASL* mutation, *GBE1* mutation, *AGL* mutation, exome sequencing

## Abstract

**Background and Objectives:** Inherited metabolic disorders (IMDs) are a group of genetic disorders characterized by defects in enzymes or transport proteins involved in metabolic processes. These defects result in an abnormal accumulation of metabolites and thus interfere with the body’s metabolism. A variety of IMDs exist and differential diagnosis is often challenging. Our objective was to gain insight into the genetic basis of IMDs and the correlations between specific genetic mutations and clinical presentations in patients admitted at various hospitals in the Madinah region of the Kingdom of Saudi Arabia. **Material and Methods:** Whole exome sequencing (WES) has emerged as a powerful tool for diagnosing IMDs and allows for the identification of disease-causing genetic mutations in individuals suspected of IMDs. This ensures accurate diagnosis and appropriate management. WES was performed in four families with multiple individuals showing clinical presentation of IMDs. Validation of the variants identified through WES was conducted using Sanger sequencing. Furthermore, various computational analyses were employed to uncover the disease gene co-expression and metabolic pathways. **Results:** Exome variant data analysis revealed missense variants in the *BTD* (c.1270G > C), *ASL* (c.1300G > T), *GBE1* (c.985T > G) and *AGL* (c.113C > G) genes. Mutations in these genes are known to cause IMDs. **Conclusions:** Thus, our data showed that exome sequencing, in conjunction with clinical and biochemical characteristics and pathological hallmarks, could deliver an accurate and high-throughput outcome for the diagnosis and sub-typing of IMDs. Overall, our findings emphasize that the integration of WES with clinical and pathological information has the potential to improve the diagnosis and understanding of IMDs and related disorders, ultimately benefiting patients and the medical community.

## 1. Introduction

Hereditary metabolic disorders or inborn errors of metabolism (IEM) are any errors or dysregulation in the metabolic pathways that are involved in homeostasis and body development [1]. These disorders are caused by genetic mutations that affect the function of enzymes and other proteins involved in various metabolic pathways. Metabolic disorders are becoming increasingly prevalent across genetically diverse populations worldwide, and they pose an increasing economic burden on public health systems in both developing and developed countries [2,3]. The global rise in the incidence of metabolic diseases is linked to several factors, including changes in dietary patterns, sedentary lifestyles, aging populations, and genetic predisposition [3,4]. Metabolic disorders can have a significant impact on the mental health of patients, which in turn can affect their productivity and quality of life. The chronic nature of metabolic disorders and the associated health problems can lead to anxiety, depression, and other mental health issues [2,5]. To mitigate the long-term effects of metabolic disorders, healthcare providers rely on a wide range of interventions. These interventions may include lifestyle changes, such as changes in diet and exercise habits, as well as medication to manage symptoms and prevent further health complications [6,7]. In recent years, the development of high-resolution molecular tools has allowed for a more accurate detection and diagnosis of metabolic disorders by examining the leading and lagging indicators in patients. For example, genetic testing can identify specific genetic variants associated with metabolic disorders, allowing for earlier diagnosis and personalized treatment plans [8,9]. 

IMDs are caused by genetic mutations that affect the enzymes involved in metabolism. The overall estimated IMD incidence is 1 case per 20,000–43,000 live births. There are many recognized types of IMDs, each with its own unique characteristics and symptoms. Most of them are inherited in an autosomal recessive manner, except for one X-linked IMDIX subtype [10]. Currently, IMDs are detected in newborns using tandem MS/MS, GC-MS, and Amino acid analyzer. In addition to metabolic tests available to date in clinical settings [11], genetic testing is often used to diagnose metabolic disorders, which typically involves blood samples from the affected individuals. This process provides a sample of DNA that can be sequenced and analyzed for genetic mutations. Genetic testing has multiple applications in both medical and non-medical settings. These tests can be employed for diagnostic purposes, carrier testing, prenatal diagnosis, newborn screening, preimplantation testing, predictive diagnosis, and pharmacogenetics testing [12,13]. Identifying specific genetic variants associated with metabolic disorders is an important step in the initial screening and diagnosis process. In the current study, using WES technology, we have identified four different genetic variants in *BTD*, *ASL*, *GBE1* and *AGL* in four Saudi families as an underlying cause of metabolic disorders. By exploring clinical manifestations and biochemical pathways, we can gain a better understanding of the commonalities and interconnections between these metabolic disorders. This knowledge can help in identifying potential diagnostic and therapeutic approaches, as well as in providing comprehensive care for the individuals affected by these conditions. 

## 2. Materials and Methods

### 2.1. Ethical Approval and Family Selection

**Figure 1:**
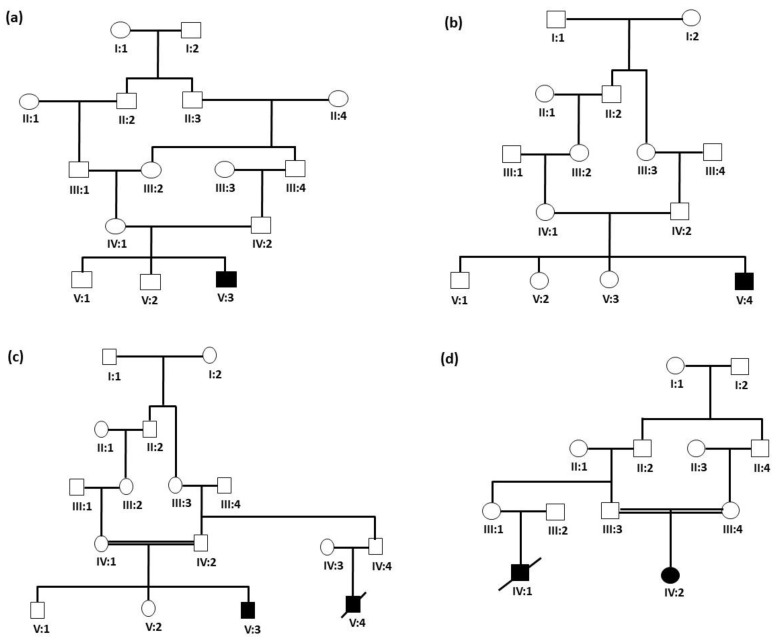
Pedigree of four Saudi families recruited for this study segregating *BTD* (**a**), *ASL* (**b**), *GBE1* (**c**) and *AGL* (**d**) mutations. Double lines show consanguineous union. Squares and circles denote males and females, respectively. Solid symbols with crossed lines represent deceased individuals. Filled and unfilled symbols represent affected individuals and unaffected individuals, respectively.

This study was carried out in accordance with all necessary guidelines provided by the research ethics committee. Ethical approval (TU-21-031, 10-05-2022) was obtained from the Ethical Research Committee of Taibah University, Madinah, Kingdom of Saudi Arabia. Four families were recruited for this study after obtaining written consent and family pedigree was drawn for the respective families based on information collected from the respective parents. Family 1 was a 5-generation family (Figure 1a) comprising 5 members, including with an affected proband (V:3), normal parents (IV:1 and IV:2) and normal siblings (V:1 and V:2). Family 2 was also a 5-generation family (Figure 1b) comprising 6 members, including an affected proband (V:4), normal siblings (V:1, V:2, V:3) and unaffected parents (IV:1 and IV:2). Family 3 was also a 5-generation family (Figure 1c) comprising 5 family members, including one affected proband (V:3), unaffected siblings (V:1, V:2) and unaffected parents (IV:1 and IV:2). Family 4 was a 4-generation family (Figure 1d) comprising 3 members, including one affected proband (IV:2) with unaffected parents (III:3 and III:4). Radiographs, CT scan, and echocardiography were performed for almost all affected individuals.

### 2.2. Whole Exome Sequencing

Genomic DNA was extracted from the blood sample of the patient. All exon regions of all human genes (approximately 23,000) were captured using xGen Exome Research Panel v2 (Integrated DNA Technologies, Coralville, IA, USA). The captured regions of the genome were sequenced with Novaseq 67,000 instrument (Illumina, San Diego, CA, USA). The raw genome sequencing data analysis, including alignment to the GRCh37/hg19 human reference genome, variant calling, and annotation, was conducted with open-source bioinformatics tools and in-house software. The variant interpretation software was developed in-house to prioritize variants based on the phenotype of each patient and ACMG guidelines. Variant filtration, classification, and similarity scoring for patient’s phenotype were performed. gnomAD (http://gnomad.broadinstitute.org/) (accessed on 7 November 2023) was used as a population genome database for estimating allele frequencies. Common variants with a minor allele frequency of >5% were filtered out in accordance with the BA1 criterion of the ACMG guidelines [14]. This step was followed by the extraction of data related to the pathogenicity of variants from a number of scientific literature sources and disease databases including ClinVar (https://www.ncbi.nlm.nih.gov/clinvar/) and UniProt (https://www.uniprot.org/) (accessed on 7 November 2023). The pathogenicity of each variant was evaluated according to the recommendations of ACMG guidelines [14]. Finally, the patient’s clinical phenotypes were transformed to corresponding standardized human phenotype ontology terms (https://hpo.jax.org/) (accessed on 7 November 2023) and accessed to measure the similarity [15,16] with each of ~7000 rare genetic diseases (https://omim.org/ and https://www.orpha.net/consor/cgi-bin/index.php) (accessed on 7 November 2023). At the end, candidate variants were manually evaluated. 

### 2.3. Confirmation of Genomic Variant by Sanger Sequencing

The validation of exome sequencing results is an essential step to ensure the accuracy and reliability of the data obtained from the sequencing process. The WES results were confirmed as described previously [17]. After designing the primers for the relevant exons to amplify the newly discovered genetic variants, the underlying genetic variants that emerged through WES were subsequently confirmed in other family members through Sanger sequencing.

### 2.4. In Silico Analysis of Protein Sequences

In order to examine the interaction, disease gene co-expression and pathways analysis, various computational analyses were conducted. The FASTA sequences were retrieved from the UniProt database and were functionally annotated. UniProt serves as an invaluable resource for researchers, scientists and the broader scientific community by facilitating the study of proteins and their functions in various biological processes [18]. For protein–protein interaction analysis, we uploaded the proteins’ FASTA sequences of *BTD*, *ASL*, *GBE1* and *AGL* to the STRING database. The STRING database is widely used in the fields of systems biology, protein interaction analysis and network biology to investigate protein functions, pathways and their interactions in various biological processes [19]. For pathways analysis, the KEGG database was used [20]. KEGG (Kyoto Encyclopedia of Genes and Genomes) is a widely used database that provides information on biological pathways, diseases, drugs and functional annotations of genes and proteins. It is a comprehensive resource for understanding the molecular interactions and networks within cells and organisms.

## 3. Results

### 3.1. Clinical Features of Patients

Family 1: The family was referred by a child specialist at the Madinah Maternity and Children Hospital (MMCH), Almadinah Almunawwarah. An affected male proband who was 49 days old with a body weight of 2 kg (V:3) (Figure 1a) was brought to the NICU of the Al-Dar hospital in Almadinah Almunawwarah. The chief clinical complaints were generalized hypertonia, poor feeding, poor activity, altered level of consciousness, recurrent hypoglycemia and extended bleeding profile. The patient was clinically evaluated with multiple CT scans of the brain followed by an X-ray and CT scan of the abdomen. The CT scan of the brain showed a large left upper parietal cortical-subcortical area with a low attenuation value, which suggested an infarct. An X-ray of the abdomen showed a gassy, swollen belly with two air-fluid levels visible on the left side. The injected contrast traveled freely via the nasogastric tube down to the rectum during a CT scan of the abdomen and pelvis with oral contrast, with no delays, obstructions or leakage as per the medical record of the patient. 

Family 2: The family directly approached the Center for Genetics and Inherited Diseases (CGID), Almadinah Almunawwarah, for the genetic evaluation of their newborn son, the affected proband (V:4) (Figure 1b), with a body weight of 2 kg at the time of birth. The proband was 2 weeks old at the time of recruitment. Blood tests revealed high levels of ammonia. High levels of ammonia (124 µmol/L) in blood may characterize other disorders such as organic acidemias, congenital lactic acidosis and fatty acid oxidation disorders, and are also present in other urea cycle disorders. Therefore, the blood sample from this patient was subjected to newborn metabolic screening. The proband was screened for thyroid stimulating hormone (TSH), Galactose-1-Phosphate Uridyltransferase (GALT), 17-hydroxyprogesterone (N17P) and biotinidase (BTD) and was found normal. However, the Argininosuccinic aciduria (ASA) screening test showed high values, depicting an increased risk for Argininosuccinic aciduria (ASA). 

Family 3: The family was referred to the Madinah Maternity and Children Hospital (MMCH), Almadinah Almunawwarah, from the remote region of Khyber, with an affected 6-month-old male proband (V:3) (Figure 1c) with chief complaints of persistent fever and cough. He was clinically evaluated and found to have mild shortening of proximal extremities with dysmorphic facial features. A skeletal X-ray and abdomen ultrasound for hepatic and renal assessment were performed. Fundoscopy was recommended for retinal evaluation. Moreover, CT scans and MRI for brain imaging were also carried out. The first-degree cousin of the proband also had similar clinical conditions and did not survive his first trimester.

Family 4: The family was also referred by a child specialist at the Madinah Maternity and Children Hospital, Almadinah Almunawwarah. The affected proband (IV:2) (Figure 1d) was a 2-month-old baby girl with chief complaints of sepsis, cardiomegaly, cardiomyopathy and lactic acidosis, along with hepatomegaly. Blood analysis revealed increased transaminases and hyperlipidemia, as well as recurrent hypoglycemia in infanthood. The proband was born to consanguine parents with one cousin with the same clinical presentation, who died at the age of one year (Figure 1d).

### 3.2. WES Identified Genetic Defects in Each Family

#### 3.2.1. Identification of Homozygous Variant in the *BTD* Gene

A likely pathogenic variant was identified in the *BTD* gene in family 1, which was found to be relevant to the patient’s phenotype, including hypertonia and hypoglycemia. The variant identified was homozygous [3-15686693-G-C; NM_001281723.3: c.1270G > C (p. Asp424His)]. Overall, population frequency was noted with an allele frequency of 0.028535. The variant was in a well-established functional domain or exonic hotspot, where pathogenic variants have frequently been reported. The p. Asp424His variant has previously been reported in Asian [21] and Brazilian populations [22]. Biotinidase deficiency is associated with the *BTD* gene, which is an autosomal recessive disorder [23]. A missense variant is a common mechanism associated with Biotinidase deficiency, and the rate of benign missense variants is relatively low [24]. In silico prediction tools and conservation analysis predicted that this variant is probably damaging to the protein structure/function (REVEL: 0.769 ≥ 0.6, 3CNET: 0.945 ≥ 0.75). An amino acid change identical to the known pathogenic variant has previously been reported with established evidence (ClinVarID: VCV000001900). 

#### 3.2.2. Identification of Homozygous Missense Variant in the *ASL* Gene

A homozygous missense variant in ASL (ASL: 7-66092817-G-T; NM_000048.3: c.1300G > T; p. Val434Leu) was identified in a patient from family 2. This variant is classified as likely pathogenic according to ACMG/AMP guidelines.

#### 3.2.3. Identification of Homozygous Variant in the *GBE1* Gene 

A missense variant [3-81691938-T-C; NM_000158.3: c.986A > G (p.Tyr329Cys)] was identified in a patient from family 3. Overall, the population allele frequency for this variant is 0.00029. The variant is in a well-established functional domain, where pathogenic variants have frequently been reported to cause autosomal recessive glycogen storage disease IV (GSDIV). The homozygous variant c.986A > G in GBE1 changes an amino acid Tyr to Cys at codon 329. Regardless of the nucleotide change reported as an established pathogenic variant, the same amino acid change has been shown to cause GSDIV (VCV000002777). It has been reported with an extremely low frequency in large population cohorts (GenomAD). Different amino acid changes in the same amino acid position have been identified as a pathogenic variant associated with this disease [25,26,27]. Multiple lines of computational evidence predict that this variant is probably damaging to the protein structure, function, or protein–protein interaction. The pathogenic variant identified in the *GBE1* gene is relevant to the patient’s phenotype, including unexplained progressive neurogenic bladder, gait difficulties from mixed upper and lower motor neuron involvement, sensory loss, predominantly in the distal lower extremities, autonomic dysfunction and mild cognitive difficulties [28,29]. 

#### 3.2.4. Identification of Homozygous Missense Variant in the *AGL* Gene

The variant [AGL; 1-99861533-C-G; NM_000028.2; c.113C > G; p.Thr38Ser] was found in the *AGL* gene of family 4 and had an overall population frequency of 0% and was classified as a missense variant. Although all in silico tools predicted this variant as benign and Varsome (https://varsome.com/variant/hg38/AGL) (accessed on 7 November 2023) classified it as a variant of unknown significance, we considered this to be disease-causing due to its rarity in the population and its segregation with phenotype in the family. 

#### 3.2.5. Confirmation of Variants’ Segregation 

**Figure 2:**
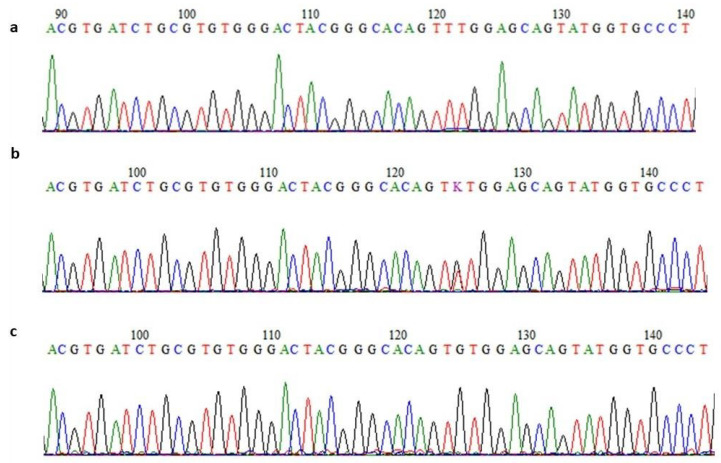
Partial amino acid sequence of *ASL* gene. Sequence chromatograms of affected (**a**), carrier (**b**) and parents (**c**) show homozygous mutant, heterozygous carrier and wild-type sequences, respectively.

The affected individuals’ exome-discovered variant mutations were confirmed by Sanger sequencing. Moreover, in order to check the segregation of identified variants, respective exons of the *BTD*, *ASL*, *GBE1* and *AGL* genes were sequenced using the Sanger method in all available DNA samples from the family members. A representative chromatogram for the *ASL* gene is shown in Figure 2. All affected individuals were homozygous for the variants while parents were heterozygous for the variants. Unaffected individuals were either heterozygous or had wild-type sequences. 

### 3.3. In Silico Gene Co-Expression and Functional Interpretations

The disease gene co-expression and pathways analysis showed coordinated expression patterns specifically in the context of inherited metabolic diseases as a pathological condition. Overall, it has been noted that genes and their sequenced variant have an association with various diseases and are diagnosed in patients suffering from several diseases. Extensive in silico analysis revealed that *BTD*, *ASL*, *GBE1* and *AGL* are the proteins involved in the pathways of biosynthesis of secondary metabolites (Table 1) and Table S1, Supplementary file). 

Secondary metabolites are considered to be the end products of primary metabolism because they are derived from the pathways in which the primary metabolites are involved, like antibiotics, toxins, pheromones and enzyme inhibitors [30]. We used network enrichment analysis or functional enrichment analysis to investigate the involvement of proteins in various diseases and metabolic pathways, utilizing STRING software (version 12.0) to predict the functional coordination of four proteins. The results revealed an interconnected role for all four proteins in metabolic pathways of the body, inherited metabolic disorders and hepatomegaly. Metabolic pathways are the only pathways in which the role of four genes (*BTD*, *ASL*, *GBE1* and *AGL*) is predicted. Moreover, functional enrichment analysis by STRING showed that *BTD*, *ASL*, *GBE1* and *AGL* are among 726 genes co-expressed during inherited metabolic disorder. *ASL*, *AGL* and *GBE1* are among 135 genes revealed to be co-expressed in carbohydrate metabolic disorder (Table S1, Supplementary file). Phenotypically, computational analysis showed that all four proteins are linked with hepatomegaly, leukemia, and heart and blood vessel abnormalities [31]. The pathogenicity prediction of variants identified in the *BTD*, *ASL*, *GBE1* and *AGL* genes using in silico tools is depicted in Table 2. 

## 4. Discussion

Patients with pathogenic mutations in the *BTD* gene present with brain imaging abnormality, decreased biotinidase levels, diffuse cerebellar atrophy, alcoholism, psychomotor retardation, infantile spasms, focal motor seizure, dyssynergia, metabolic ketoacidosis, limb muscle weakness, propionyl-CoA carboxylase deficiency, lactic acidosis, developmental regression, spastic paraparesis, myelopathy, basal ganglia calcification, generalized myoclonic seizure, bilateral tonic–clonic seizure, organic aciduria, hyperammonemia, acidosis, generalized hypotonia, global developmental delay, coma, lethargy, muscular hypotonia and ataxia [32,33]. The B-complex vitamin biotin is utilized by the body with the help of the enzyme biotinidase (BTD). Biotinidase deficiency causes the body to improperly recycle the vitamin biotin [34]. Biotinidase deficiency with similar clinical features has not been reported before in the Middle East region, especially in Saudi Arabia. In this study, we reported the clinical and genetic presentation of a 49-day-old male pediatric case. The second disease studied here is argininosuccinate lyase (ASL) deficiency. ASA is a rare inherited autosomal recessive metabolic disorder that affects the urea cycle, a metabolic pathway responsible for converting toxic ammonia into urea for excretion from the body [35,36,37]. ASL is one of the enzymes involved in the urea cycle and plays a crucial role in breaking down argininosuccinic acid into arginine and fumarate. Argininosuccinate lyase deficiency (ASLD) can present in two distinct forms: severe neonatal-onset and late-onset [36,38]. In the severe neonatal-onset form, infants typically develop symptoms of hyperammonemia, a condition characterized by elevated levels of ammonia in the blood, within the first few days after birth. The prevalence of ASLD is estimated to be approximately 1 in 70,000 live births, making it the second most common urea cycle disorder after ornithine transcarbamylase deficiency (OTCD). Although ASL and argininosuccinate synthase (ASS1) are part of the urea cycle in the liver, they are also expressed in most tissues outside the liver. It is possible that their expression in various tissues is driven by the arginine synthesis requirement for other metabolic cycles. For example, NO synthesis uses arginine as a substrate. ASL and ASS1 are key players in the citrulline–NO cycle, in which citrulline is regenerated from arginine for the synthesis of NO [39]. Blood tests may also reveal high levels of ammonia. High levels of ammonia in blood may characterize other disorders such as organic acidemias, congenital lactic acidosis and fatty acid oxidation disorders, and are also present in other urea cycle disorders. Therefore, we used metabolic screening and genetic testing to confirm the diagnosis.

The third family studied had a mutation in the *GBE1* gene, which encodes the glycogen branching enzyme. Glycogen storage disease IV is associated with *GBE1* gene mutations, which is an autosomal recessive disorder. Patients affected by Glycogen storage disease IV present with hepatic failure, peripheral neuropathy, tubulointerstitial fibrosis, hyperlordosis, limb joint contracture and arthrogryposis multiplex congenital [40]. The variant (p.Tyr329Cys) identified in the patient is predicted to be damaging by several computational analyses. Family 4 in this study has a mutation in the *AGL* gene. Mutations in AGL are associated with glycogen storage disease IIIa and IIIb, which lead to amylo-1,6-glucosidase deficiency, hypoglycemia, hyperlipidemia, elevated transaminases, and increased serum creatine kinase, but normal blood lactate and uric acid [41]. The glycogen storage disease IIIa has been reported to be caused by various variants such as splice site variants (IVS6 + 1G > A, IVS6-1G > A, IVS14 + 1G > T, IVS26-2A > C), nonsense mutations (R469X, R864X, S929X, R977X, Y1428X), small deletions (c.408-411delTTTG, c.2717-2721delAGATC, c.2823delT) and insertion (c.4234insT) mutations [42,43,44]. The identification of the novel variant (c.113C > G; p.T38S) in this study has been submitted to ClinVar (RCV000723338) as a variant of unknown significance (VUS). Recurrent submissions of VUS to databases help in the classification of variants as well as their association with clinical phenotypes (phenotype–genotype correlation) (Table S2, Supplementary file). Mutations in the *AGL* gene lead to defective amyloid-1,6-glucosidase, the primary glycogen debranching enzyme, subsequently causing decreased glycogen degradation and an accumulation of limited dextrin in the affected organs (primarily skeletal muscle, cardiac muscle and liver). Moreover, it leads to organomegaly and the dysfunction of several vital organs. Glycogen storage disease type IIIb exclusively affects the liver; however, glycogen storage disease type IIIa affects the heart, skeletal and liver muscles [45]. WES presents advantages over single gene or panel tests and WGS [46]. By sequencing the protein-coding exonic regions of the human genome, WES provides more genetic information than targeting a selected set of genes, while also being more cost-effective and time-efficient than WGS. This makes WES more insightful for patients harboring diseases of uncertain or heterogeneous genetic origin and increases accessibility to clinical genetic testing [47]. As with many scientific methods, WES possesses certain limitations. For instance, it does not encompass the entire genome, and its validation is restricted to the detection of structural variation, including CNVs, inversions, and translocations. Furthermore, it fails to sequence non-coding intron regions and repeat sequences [47]. We suggest the use of WES for genetic diagnosis in heterogeneous metabolic disorders. WES not only aids in exact diagnosis, but also helps in the early diagnosis and management of patients.

## 5. Conclusions

In conclusion, we analyzed various families clinically and genetically, and identified variants in genes involved in metabolic pathways. Variants in *BTD* (c.1270G > C; p. Asp424His); *ASL* (c.1300G > T; p.Val434Leu); *GBE1* (c.985A > G; p.Tyr329Cys); and *AGL* (c.113C > G; p.T38S) have broadened the spectrum of mutations in these genes and provided additional evidence of involvement of these genes, when mutated, in various metabolic diseases. Moreover, various extensive computational analyses revealed that all four genes are involved in vital metabolic pathways. Briefly, the product of these genes encodes enzymes that play vital roles in different metabolic pathways, and when mutated, causes inherited metabolic diseases including glycogen storage disease, biotinidase deficiency and Argininosuccinic aciduria. This knowledge could be helpful in identifying potential diagnostic and therapeutic approaches, as well as in providing comprehensive care for the individuals affected by these conditions. 

## Figures and Tables

**Table 1:** Computational analysis of *BTD*, *ASL*, *GBE1* and *AGL* genes.

Name of Gene	Protein Family	Domain	Pathways
Biotinidase (***BTD***)	Biotinidase-like, eukaryotic, Biotinidase/VNN family	Carbon-nitrogen hydrolase, Vanin, C-terminal	Biotin metabolism. Metabolic pathways. Vitamin digestion and absorption.
Arginosuccinate lyase (***ASL***)	Fumarate lyase family Argininosuccinate lyase	Fumerate_Lyase_N/Argininosuccinate Lyase	Arginine biosynthesis. Alanine, aspartate, and glutamate metabolism. Metabolic pathways. Biosynthesis of secondary metabolites. Biosynthesis of amino acids.
Glucan-branching enzyme (***GBE1***)	GlgB 1,4-alpha-glucan-branching enzyme	Glycoside hydrolase, family 13, N-terminal Alpha-amylase	Starch and sucrose metabolism. Metabolic pathways. Biosynthesis of secondary metabolites.
Glycogen Debranching Enzyme (***AGL***)	Glycogen debranching enzyme, metazoa	Glucanotransferase, Glycogen debranching enzyme, C-terminal	Starch and sucrose metabolism. Metabolic pathways. Biosynthesis of secondary metabolites.

**Table 2:** Pathogenicity prediction of variants identified in *BTD*, *ASL*, *GBE1* and *AGL* genes using in silico tools.

Variant Predictions	*BTD* (c.1270G > C)	*ASL* (c.1300G > T)	*GBE1* (c.985T > G)	*AGL* (c.113C > G)
Protein change	p. Asp424His	p. Val434Leu	p.Tyr329Cys	p.Thr38Ser
ACMG classification	Pathogenic	Likely Pathogenic	Pathogenic	Likely Benign
PhyloP score	3.238	8.539	8.803	4.125
REVEL	Pathogenic	Pathogenic	Pathogenic	Benign
SIFT	Uncertain	Uncertain	Pathogenic	Benign
MutationTaster	Benign	Uncertain	Uncertain	Benign
MutationAssesor	Pathogenic	Benign	Pathogenic	Benign
Provean	Pathogenic	Uncertain	Pathogenic	Benign

## Data Availability

All data are available upon request.

## Supplementary Materials

The following supporting information can be downloaded at: https://www.mdpi.com/article/10.3390/jcm13051193/s1, Table S1: Functional enrichment analysis of *BTD*, *ASL*, *GBE1* and *AGL* genes. Table S2: Comparison of clinical phenotypes of our patients with previously published reports.
