# Supplementary material for: Identification of Novel and Recurrent Variants in BTD, GBE1, AGL and ASL Genes in Families with Metabolic Disorders in Saudi Arabia
Source: J Clin Med. 2024 Feb 20;13(5):1193. doi: 10.3390/jcm13051193 (PMC10932034; doi:10.3390/jcm13051193)
Supplement: Supplementary file 1 [file jcm-13-01193-s001.zip › jcm-2689618-supplementary.pdf]

Identification of Novel and Recurrent Variants in *BTD, GBE1, AGL* and *ASL* Genes in Families with Metabolic Disorders in Saudi Arabia

Muhammad Latif [1,2,*,†], Jamil Amjad Hashmi [1,2,†], Abdulfatah M. Alayoubi [1], Arusha Ayub [3] and Sulman Basit [1,2,*]

[1] Department of Basic Medical Sciences, College of Medicine, Taibah University, Medina 42353, Saudi Arabia; jamjadali@taibahu.edu.sa (J.A.H.); aayoubi@taibahu.edu.sa (A.M.A.)
[2] Center for Genetics and Inherited Diseases, Taibah University, Medina 42353, Saudi Arabia; mmuradkhan@taibhau.edu.sa (M.L.); sbasit.phd@gmail.com (S.B.)
[3] Department of Medicine, School of Health Sciences, University of Georgia, Athens, GA 30602, USA; arushaayub.std@ug.edu.ge (A.A.)
[*] Correspondence: latifmayo@gmail.com (M.L.); sbasit.phd@gmail.com (S.B.); Tel.: +966-14-8618888 (Ext77596) (M.L.); +966-53-5370-209 (S.B.)
[†] These authors contributed equally to this work.

Table S1. Functional enrichment analysis of *BTD, ASL, GBE1,* and *AGL* genes

**Local network cluster (STRING)**				
Cluster	*Description*	*Count in Network*	*Strength*	*False Discovery rate*
CL:11741	Glycogen metabolism	2 of 19	2.71	0.0299
**KEGG Pathways**				
Pathway	*Description*	*Count in Network.*	*Strength*	*False Discovery rate*
hsa00500	Starch and sucrose metabolism	2 of 32	2.49	0.0059
hsao1100	Metabolic pathways	4 of 1447	1.13	0.0059
**Reactome Pathway**				
Pathway	*Description*	*Count in Network*	*Strength*	*False Discovery rate*
HSA 8982491	Glycogen metabolism	2 of 27	2.56	0.0276
**Disease gene associations (Diseases)**				
Disease	*Description*	*Count in Network*	*Strength*	*False Discovery rate*
0010:655	Inherited metabolic disorder	4 of 726	1.43	0.0060
0010:2747	Glycogen storage disease	2 of 28	2.54	0.0149
0010:2978	Carbohydrate metabolic disorder	3 of 135	2.04	0.0060
**Human Phenotype (Monarch)**				
Phenotype	*Description*	*Count in Network*	*Strength*	*False Discovery rate*
HP:0002240	Hepatomegaly	4 of 544	1.56	0.0060
HP:0002910	Elevated hepatic transaminase	3 of 233	1.8	0.0170

Table S2: Comparison of clinical phenotypes of our patients with previously published reports

Disease with variant	Other studies		Our study
**BTD (c.1270G>C); (p. Asp424His)**	**Wolf et al. 1985,** develop seizures, hypotonia, skin rash, alopecia, and developmental delay with keto-lactic acidosis and organic aciduria, ataxia, conjunctivitis, optic atrophy, progressive hearing loss, and occasionally coma	**Liu et al. 2017,** Fatigue, hypotonia, proximal muscular weakness, hearing deficits, rash, and respiratory problems are common clinical phenotype	Hypertonia, poor feeding, poor activity, altered level of consciousness, recurrent hypoglycemia, and extended bleeding profile
**ASL (c.1300G>T); (p.Val434Leu)**	**Bijarnia-Mahay et al. 2018**, Neonatal encephalopathy, convulsions, lethargy, reduced feeding		Poor feeding, lethargic, seizures, newborn metabolic screening reports showing high ASA.
**GBE1 (c.986A>G (p.Tyr329Cys)**	**Nair et al. 2018,** Failure to thrive; hepatosplenomegaly; muscle weakness (Neurological)		Persistent fever and cough, mild shortening of proximal extremities with dysmorphic facial features, unexplained progressive neurogenic bladder, gait difficulties from mixed upper and lower motor neuron involvement, sensory loss predominantly in the distal lower extremities, autonomic dysfunction, and mild cognitive difficulties
**AGL (c.113C>G (p.Thr38Ser)**	No published literature is available		Sepsis, cardiomegaly, cardiomyopathy, lactic acidosis along with hepatomegaly. Blood analysis revealed increased transaminases and hyperlipidemia, as well as recurrent hypoglycemia in childhood.
